# Complete Genome sequence of the nematicidal *Bacillus thuringiensis* MYBT18246

**DOI:** 10.1186/s40793-017-0259-x

**Published:** 2017-08-24

**Authors:** Jacqueline Hollensteiner, Anja Poehlein, Cathrin Spröer, Boyke Bunk, Anna E. Sheppard, Philip Rosentstiel, Hinrich Schulenburg, Heiko Liesegang

**Affiliations:** 1 Department of Genomic and Applied Microbiology & Göttingen Genomics Laboratory, Institute of Microbiology and Genetics, University of Göttingen, Göttingen, Germany; 2 Leibniz Institute DSMZ-German Collection of Microorganisms and Cell Cultures, Braunschweig, Germany; 3 Present address: Nuffield Department of Medicine, University of Oxford, Oxford, UK; 4 Department of Evolutionary Ecology and Genetics, Zoological Institute, Christian-Albrechts University of Kiel, Kiel, Germany; 5 Institute of Clinical Molecular Biology, Christian-Albrechts University of Kiel, Kiel, Germany

**Keywords:** *Bacillus thuringiensis*, *Bacillus cereus* sensu *lato*, Prophages, Parasporal crystal protein, Pan-Core-genome

## Abstract

10.1601/nm.5000 is a rod-shaped facultative anaerobic spore forming bacterium of the genus 10.1601/nm.4857. The defining feature of the species is the ability to produce parasporal crystal inclusion bodies, consisting of δ-endotoxins, encoded by *cry*-genes. Here we present the complete annotated genome sequence of the nematicidal 10.1601/nm.5000 strain MYBT18246. The genome comprises one 5,867,749 bp chromosome and 11 plasmids which vary in size from 6330 bp to 150,790 bp. The chromosome contains 6092 protein-coding and 150 RNA genes, including 36 rRNA genes. The plasmids encode 997 proteins and 4 t-RNA’s. Analysis of the genome revealed a large number of mobile elements involved in genome plasticity including 11 plasmids and 16 chromosomal prophages. Three different nematicidal toxin genes were identified and classified according to the Cry toxin naming committee as *cry*13Aa2, *cry*13Ba1, and *cry*13Ab1. Strikingly, these genes are located on the chromosome in close proximity to three separate prophages. Moreover, four putative toxin genes of different toxin classes were identified on the plasmids p120510 (Vip-like toxin), p120416 (Cry-like toxin) and p109822 (two Bin-like toxins). A comparative genome analysis of 10.1601/nm.5000 MYBT18246 with three closely related 10.1601/nm.5000 strains enabled determination of the pan-genome of 10.1601/nm.5000 MYBT18246, revealing a large number of singletons, mostly represented by phage genes, morons and cryptic genes.

## Introduction

10.1601/nm.5000 is an ubiquitously distributed, rod-shaped, Gram-positive, spore forming, facultative anaerobic bacterium [1, 2]. 10.1601/nm.5000 has been isolated from various ecological niches, including soil, aquatic habitats, phylloplane and insects [3–7]. The defining property of the species is the ability to produce parasporal protein crystals consisting of δ-endotoxins, which are predominantly encoded on plasmids [1, 8, 9]. These proteins are toxic towards a wide spectrum of invertebrates of the orders *Lepidoptera* *,* *Diptera* *,* *Coleoptera* *,* *Hymenoptera* *, Homoptera,* *Orthoptera* *, Mallophaga* and other species like *Gastropoda*, mites, protozoa and especially nematodes [7, 10–12]. In addition, 10.1601/nm.5000 produce additional toxins such as Cyt, Vip, and Sip toxins [13]. Cry toxins represent the largest group and can be subdivided into three different homology groups. In total, over 787 different Cry toxins have been identified, each exhibiting toxicity against a specific host organism [14]. It has been shown that 10.1601/nm.5000 strains can produce more than one Cry toxin resulting in a broad host range. As such, 10.1601/nm.5000 has been used widely as a biopesticide in agriculture for several decades [1, 2, 8, 13, 15, 16]. 10.1601/nm.5000 is a member of the genus 10.1601/nm.4857, which are low GC-content, Gram-positive bacteria with a respiratory metabolism and the ability to form heat- and desiccation-resistant endospores [11, 17, 18]. Within this genus, 10.1601/nm.5000 is a member of the 10.1601/nm.4885 sensu *lato* species group which originally contained seven different species (10.1601/nm.4885, 10.1601/nm.4871 *,* 10.1601/nm.5000 *,* 10.1601/nm.4947 *,* 10.1601/nm.4966 *,* 10.1601/nm.5007 *,* 10.1601/nm.23711 [17–25]). Historically, most pathogenic and phenotypic properties were used for strain classification. However, recent publications utilizing genomic criteria suggest that the species group should be extended by species 10.1601/nm.24560 [26, 27]. Moreover, the three proposed species *“* 10.1601/nm.27585”[28]*, *“10.1601/nm.27575”[29] and *“* 10.1601/nm.26046” [30] have been isolated and effectively published. However, these names had not yet appeared on a Validation List at the time of pulbication [31]*.* Due to the very close phylogenetic relationships, it has also been proposed to assign the eleven species to a single extended Bcsl species [32, 33]. The genome of Bcsl-members contains a highly conserved chromosome with regard to gene content, sequence similarity and genome synteny, while variation can be observed within mobile genomic elements such as prophages, insertion elements, transposons, and plasmids [34]. Due to the significance of Bcsl group members in human health, the food industry and agriculture, resolving the phylogeny is of great importance. Because of the highly conserved 16S rRNA-genes, the classical 16S phylogeny of Bcsl strains is inconclusive. Thus, a combination of 16S and a seven gene multi-locus sequence typing scheme have been used to establish taxonomic relationships within species of the Bcsl-group [35, 36]. Comparative genomics of the *cry*-gene loci has revealed remarkable proximity to elements of genome plasticity such as plasmids, transposons, insertion elements and prophages [2, 37–39]. The activity of these mobile elements has resulted in a magnitude of highly diverse plasmid sizes through rearrangements such as deletions and insertions, as well as migration of *cry*-genes into the bacterial chromosome [40]. The worldwide distribution of 10.1601/nm.5000 and its capacity to adapt to a diverse spectrum of invertebrate hosts is explained by the formation of spores and a remarkable variability in crystal protein families [13]. This toxin arsenal, especially the copy number of individual toxin genes, can be shaped by reciprocal co-adaptation with a nematode host, as previously demonstrated using controlled evolution experiments in the laboratory [41, 42]. The 10.1601/nm.5000 strain MYBT18246 described herein and its host *Caenorhabditis elegans* have been selected as a model system for such co-evolution experiments [41]. One aim of this sequencing project was to provide a high-quality reference genome sequence for the original 10.1601/nm.5000 MYBT18246 in order to obtain a detailed phylogeny and shed light on the evolution of this microparasite, with a particular focus on the presence of virulence factors, elements of genome plasticity and host adaptation factors. Here we present the genome of the nematicidal 10.1601/nm.5000 MYBT18246 and its comparative analysis to the three closest relatives identified by MLST phylogeny.

## Organism information

### Classification and features

**Fig. 1:**
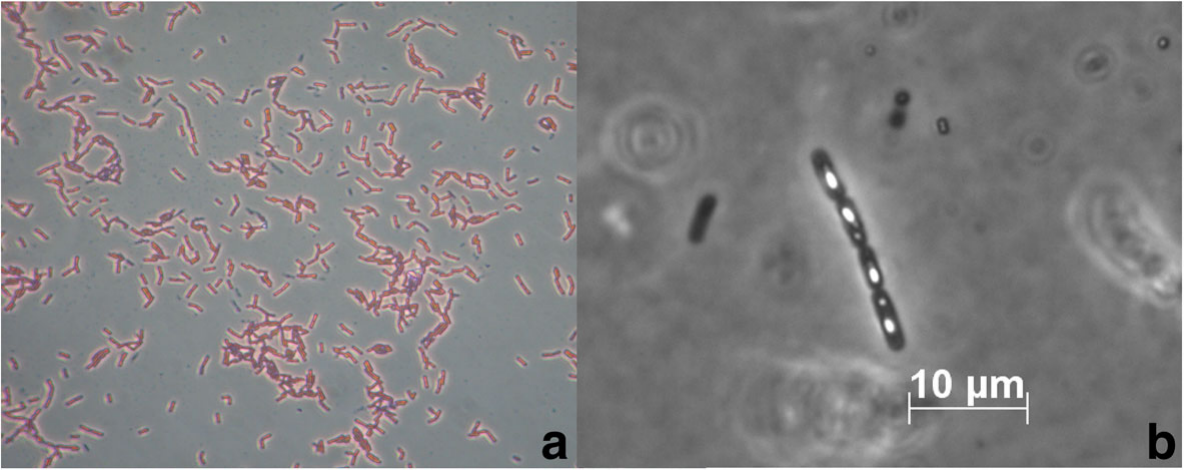
Microscopic characteristics of 10.1601/nm.5000 MYBT18246. **a** Light microscope analysis of Gram stained 10.1601/nm.5000 MYBT18246 cells (40×). **b** Phase contrast microscope analysis of sporulated and Cry-toxin producing cells of 10.1601/nm.5000 MYBT18246 (40×)

10.1601/nm.5000 belongs to the genus 10.1601/nm.4857 and has been isolated in the end of the nineteenth century [17, 20] and used as a biocontrol agent for several decades [7, 18, 21]. The strain 10.1601/nm.5000 MYBT18246 is a Gram-positive, rod-shaped and spore forming bacterium (Fig.1a), as most 10.1601/nm.5000 [7]. 10.1601/nm.5000 MYBT18246 was isolated in the Schulenburg lab by AS from a mixture of genotypes present in the strain 10.1601/strainfinder?urlappend=%3Fid%3DNRRL+B-18246, originally provided by the Agricultural Research Service Patent Culture Collection (United States Department of Agriculture, Peoria, IL, USA) [43–45]. As a member of the species 10.1601/nm.5000, 10.1601/nm.5000 MYBT18246 is facultative anaerobe, motile and is able to produce parasporal crystal toxins, which is the characteristic feature of this species [2]. Growth occurred at temperatures ranging from 10 to 48 °C and optimal growth was monitored at mesophil temperatures ranging from 28 to 37 °C [46]. The pH range of 10.1601/nm.5000 strains varies from pH 4.9 to 8.0, with the optimum documented as pH 7 [47, 48]. Strain 10.1601/nm.5000 MYBT18246 exhibits flat, opaque colonies with undulate, curled margins and produced crystals during the stationary phase (Fig. 1a-b). Characteristic features of 10.1601/nm.5000 MYBT18246 are listed in Table 1.

**Table 1:** Classification and general features of 10.1601/nm.5000 MYBT18246 [54]

MIGS ID	Property	Term	Evidence code^a^
	Classification	Domain *Bacteria*	TAS [86]
		Phylum 10.1601/nm.3874	TAS [47]
		Class 10.1601/nm.4854	TAS [87, 88]
		Order 10.1601/nm.4855	TAS [18, 89]
		Family 10.1601/nm.4856	TAS [18, 90]
		Genus 10.1601/nm.4857	TAS [17, 18]
		Species 10.1601/nm.5000	TAS [46]
		Strain MYBT18246	IDA
	Gram stain	positive	IDA
	Cell shape	rod-shaped	IDA
	Motility	Motile	TAS [46]
	Sporulation	Spore-forming	IDA
	Temperature range	10–48 °C	TAS [46]
	Optimum temperature	28–37 °C	TAS [46]
	pH range; Optimum	4.9–8.0; 7.0	TAS [47, 48]
	Carbon source	Organic carbon source	NAS
MIGS-6	Habitat	Worldwide	TAS [7]
MIGS-6.3	Salinity	Salt tolerant	TAS [7]
MIGS-22	Oxygen requirement	Aerobic, facultative anaerobic	TAS [11]
MIGS-15	Biotic relationship	Free-living, microparasite of *C. elegans*	TAS [41]
MIGS-14	Pathogenicity	Nematode pathogen	TAS [41]
MIGS-4	Geographic location	not reported	
MIGS-5	Sample collection	not reported	
MIGS-4.1	Latitude	unreported	
MIGS-4.2	Longitude	unreported	
MIGS-4.4	Altitude	unreported	

^a^Evidence codes - IDA: Inferred from Direct Assay; TAS: Traceable Author Statement (i.e., a direct report exists in the literature); NAS: Non-traceable Author Statement (i.e., not directly observed for the living, isolated sample, but based on a generally accepted property for the species, or anecdotal evidence). These evidence codes are from the Gene Ontology project

#### Extended feature descriptions

**Fig. 2:**
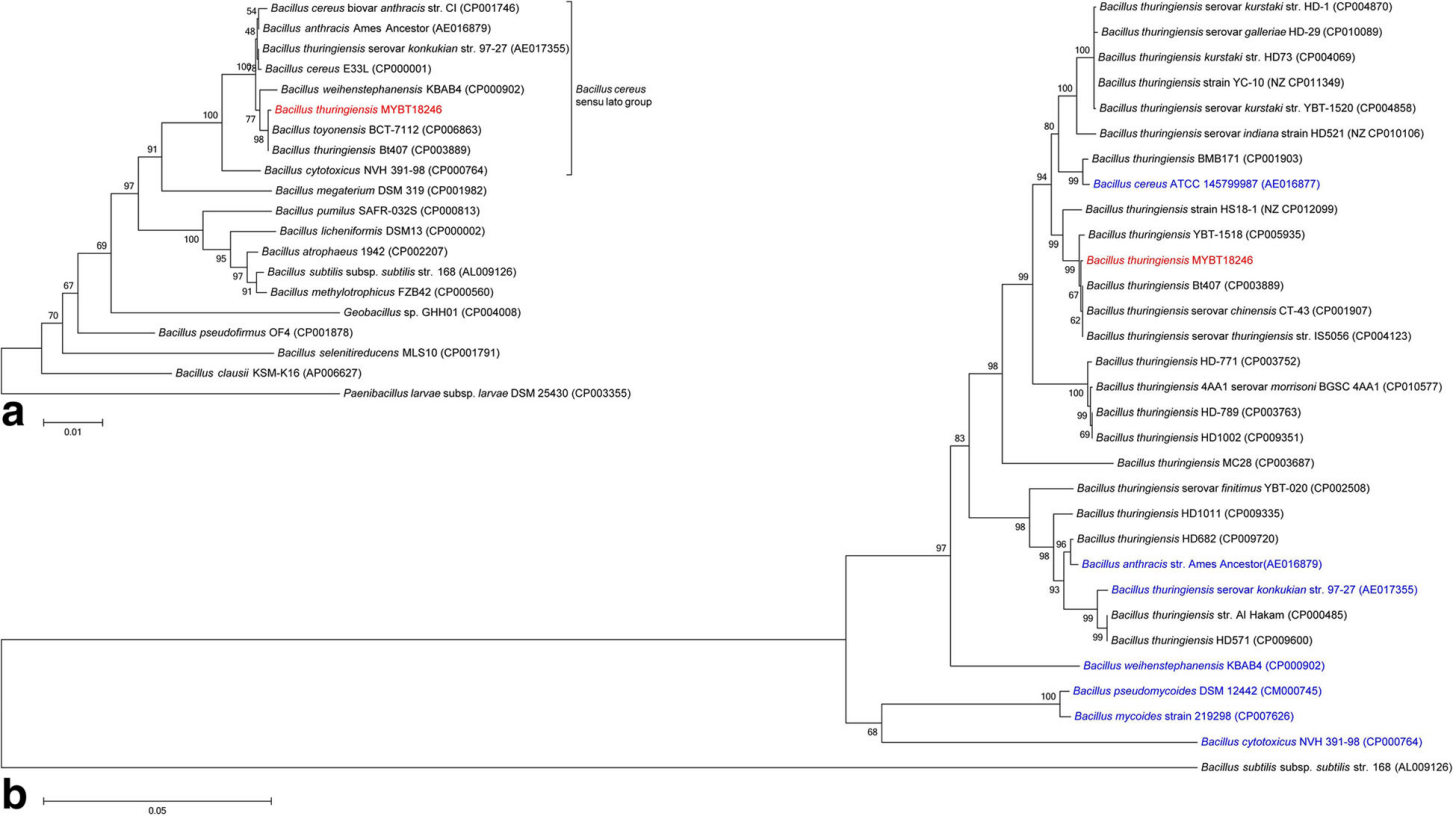
Phylogenetic tree highlighting the taxonomic relation of 10.1601/nm.5000 MYBT18246 (red) based on **a**) 16rDNA amplicon within the 10.1601/nm.4857 clade **b**) Multi-locus sequence typing within the 10.1601/nm.4885 sensu *lato* species group. GenBank accession numbers are given in parentheses. Comparison includes strains of the 10.1601/nm.4854 clade or Bcsl group members (blue). 10.1601/nm.5141 10.1601/strainfinder?urlappend=%3Fid%3DDSM+25430 or 10.1601/nm.4858 str. 168 has been used as outlier to root the tree. Sequences were aligned using ClustalW 1.6 [91, 92]. The phylogenetic tree was constructed by using the Neighbor-Joining method [50] and evolutionary distances were computed by the Maximum Composite Likelihood method [51] within MEGA7.0 [52]. Numbers at the nodes are bootstrap values calculated from 1000 replicates

The cell size of 10.1601/nm.5000 can vary from 0.5 × 1.2 μm - 2.5 × 10 μm [11]. Categorization into the group of Gram-positive organisms was confirmed by Gram staining, as shown in Fig. 1a. In Fig. 1b the production of Cry toxins can be observed. These toxins accumulate during the sporulation phase next to the endospore and build phase-bright inclusions [7]. 10.1601/nm.5000 MYBT18246 exhibited 99% 16S rRNA sequence identity to other published Bcsl*-*members [49]. As a result of the high sequence similarity, a phylogenetic differentiation of 10.1601/nm.5000 MYBT18246 based on 16S phylogenetic differentiation of Bcsl group members is impossible (Fig. 2a). As an alternative, 23 10.1601/nm.5000 strains, and a representative of each of the Bcsl group species were chosen for phylogenetic analysis using multi-locus sequence typing as previously developed by Priest [36] (Fig. 2b). 10.1601/nm.4858 str. 168 was selected as an outgroup to root the tree [17, 18]. The phylogenies were generated using the Neighbor-Joining method [50] and evolutionary distances were computed by the Maximum Composite Likelihood method [51]. In total, 217 MLST gene sequences were compared with 1000 bootstrap replicates. Phylogenetic analysis was conducted in MEGA7 [52]. All used reference sequences were retrieved from GenBank hosted at NCBI.

## Genome sequencing information

### Genome project history

10.1601/nm.5000 MYBT18246 was used in a co-evolution study with a *Caenorhabditis elegans* host. The original strain MYBT18246 was selected for sequencing in order to generate a reliable reference sequence for subsequent experiments [41, 42]. The genome sequence was analyzed to identify virulence factors and fitness factors contributing to the efficient infection of *C. elegans*. Additionally, the phylogenetic position of 10.1601/nm.5000 MYBT18246 in the Bcsl group was determined [41]. The complete genome sequence has been deposited in GenBank with the accession numbers (CP015350-CP015361) and in the integrated Microbial Genomes database with the Taxon ID 2671180122 [53]. A summary of the project information and its association with MIGS version 2.0 compliance [54] is shown in Table 2.

**Table 2:** Project information

MIGS ID	Property	Term
MIGS 31	Finishing quality	Complete
MIGS-28	Libraries used	Two genomic libraries: 454 pyrosequencing shotgun library, PacBio library
MIGS 29	Sequencing platforms	454 GS FLX system, PacBioRSII
MIGS 31.2	Fold coverage	18 × 454; 50 × PacBio
MIGS 30	Assemblers	Newbler 2.8; HGAP v2.3.0
MIGS 32	Gene calling method	Prodigal 2.6
	Locus Tag	BT246
	Genbank ID	CP015350-CP015361
	GenBank Date of Release	2016–07-15
	GOLD ID	Gp0020852
	BIOPROJECT	PRJNA290307
MIGS 13	Source Material Identifier	Department of Evolutionary Ecology and Genetics, CAU, Kiel
	Project relevance	Evolution

### Growth conditions and genomic DNA preparation

Genomic DNA was isolated from 10.1601/nm.5000 MYBT18246 using the DNeasy blood and tissue kit (Qiagen, Hilden, Germany) for 454 pyrosequencing [55] and the Genomic-Tip 100/G Kit (Qiagen, Hilden, Germany) for Single Molecule real-time sequencing [56] according to the manufacturer’s instructions. For SMRT-sequencing the procedure and Checklist: Greater than 10 kb Template Preparation Using AmPure PB Beads was used and blunt end ligation was applied overnight. Whole-genome sequencing was performed using a 454 GS-FLX system (Titanium GS70 chemistry; Roche Life Science, Mannheim, Germany) and on one SMRT Cell on the PacBio *RSII* system using P6-chemistry (Pacific Biosciences, Menlo Park, CA, USA).

### Genome sequencing and assembly

A summary of the project information can be found in Table 2. 454-pyrosequencing was carried out at the Institute of Clinical Molecular Biology in Kiel, Germany and SMRT-sequencing at the DSMZ Braunschweig. First, approximately 331,000,454-reads with an average length of 600 bp were assembled using the Newbler 2.8 de novo assembler (Roche Diagnostics), resulting in 729 contigs with a coverage of 18 x. Repeats were resolved and gaps between contigs were closed using PCR with Sanger sequencing of the products with BigDye 3.0 chemistry and an 10.1601/strainfinder?urlappend=%3Fid%3DABI+3730XL capillary sequencer (Applied Biosystems, Life Technology GmbH, Darmstadt, Germany). Manually editing in Gap4 (version 4.11) software of the Staden package [57] was performed to improve the sequence quality. For final gap closure PacBio sequencing was used. A total of 27,870 PacBio reads with a mean length of 14,053 bp were assembled using HGAP 2.0 [58], resulting in a coverage of 50 x, with further analysis using SMRT Portal (v2.3.0) [59]. Finally, both assemblies were combined, resulting in 12 contigs including a closed circular chromosome sequence of 5,867,749 bp. Eight additional contigs exhibited overlapping ends and were circularized to plasmid sequences ranging from 6.3 kb to 150 kb (Table 3). The assembly was checked for coverage drop downs and extremes of disparities including GC, AT, RY, and MK. Moreover, we determined the origin of replication of 10.1601/nm.5000 MYBT18246 by comparative analysis with OriC of eight other 10.1601/nm.5000 strains available in DoriC [60, 61]. These strains varied in chromosome size from 5.2 Mb to 5.8 Mb but all shared a similar GC-content of 35%. In total, including 10.1601/nm.5000 MYBT18246, two OriC regions were identified using the ORF-Finder [62]. One region was highly conserved with regard to OriC length (178/179 nt), OriC AT content (~0.69) and number of DnaA boxes (4). The second region varied in OriC length (564–767 nt) and OriC AT content (~0.67–0.7), but all had the same number of DnaA boxes (9). 10.1601/nm.5000 MYBT18246 showed the highest OriC similarities with both OriC regions of 10.1601/nm.5000 Bt407.

**Table 3:** Summary of genome: one chromosome and 11 plasmids

Label	Size (Mb)	Topology	INSDC identifier	RefSeq ID
Chromosome	5.8	Circular	CP015350	NZ_CP015350.1
Plasmid 1	0.151	Circular	CP015351	NZ_CP015351.1
Plasmid 2	0.142	Circular	CP015352	NZ_CP015352.1
Plasmid 3	0.121	Circular	CP015353	NZ_CP015353.1
Plasmid 4	0.120	Circular	CP015354	NZ_CP015354.1
Plasmid 5	0.110	Circular	CP015355	NZ_CP015355.1
Plasmid 6	0.101	Circular	CP015356	NZ_CP015356.1
Plasmid 7	0.055	Linear	CP015357	NZ_CP015357.1
Plasmid 8	0.047	Circular	CP015358	NZ_CP015358.1
Plasmid 9	0.017	Linear	CP015359	NZ_CP015359.1
Plasmid 10	0.014	Linear	CP015360	NZ_CP015360.1
Plasmid 11	0.006	Circular	CP015361	NZ_CP015361.1

### Genome annotation

Annotation was performed with Prokka v1.9 [63] using the manually curated 10.1601/nm.5000 strain Bt407 [64] as a species reference and a comprehensive toxin protein database (including Cry, Cyt, Vip, and Sip toxins) as feature references. The Prokka pipeline was applied using prodigal for gene calling [65]. RNAmmer 1.2 [66] and Aragorn [67] were used for rRNA gene and t-RNA identification, respectively. Additionally, signal leader peptides were identified with SignalP 4.0 [68] and non-coding RNAs with an Infernal 1.1 search against the Rfam database [69]. Annotation of *cry* toxin genes were manually corrected and named according to the standards of the Cry toxin nomenclature by Crickmore [70]. Identified toxins were deposited at the 10.1601/nm.5000 Toxin nomenclature database [14].

## Genome properties

The genome of 10.1601/nm.5000 MYBT18246 consists of 12 replicons with a circular chromosome of 5,867,749 bp (Table 3). The GC content of the chromosome is 35% and the GC content of the plasmids ranges from 32 to 37%. The total number of protein coding genes is 7089 with 6092 genes on the chromosome and 997 genes on the plasmids. The genome harbors 12 rRNA clusters, 111 t-RNA genes, 5274 predicted protein-coding genes with assigned function and 1815 genes encoding proteins with unknown function (Table 4). All gene products have been assigned to COGs (Table 5). The genome sequence of 10.1601/nm.5000 MYBT18246 is available in GenBank (CP015350 for the chromosome and CP015351 - CP015361 for the plasmids).

**Table 4:** Genome statistics

Attribute	Value	% of Total
Genome size (bp)	6,752,488	100
DNA coding (bp)	5,623,665	83.28
DNA G + C (bp)	2,389,665	35.39
DNA scaffolds	12	100
Total genes	7239	100
Protein coding genes	7089	97.9
RNA genes	151	2.09
Genes in internal clusters	2694	37.22
Genes with function prediction	5274	72.86
Genes assigned to COGs	4662	64.40
Genes with Pfam domains	5503	76.02
Genes with signal peptides	500	6.91
Genes with transmembrane helices	1863	25.74
CRISPR repeats	0	0

**Table 5:** Number of protein encoding genes associated with general COG functional categories

Code	Value	%	Description
J	226	3.19	Translation, ribosomal structure and biogenesis
A	0	0	RNA processing and modification
K	487	6.87	Transcription
L	625	8.82	Replication, recombination and repair
B	1	0.01	Chromatin structure and dynamics
D	59	0.83	Cell cycle control, Cell division, chromosome partitioning
V	141	1.99	Defense mechanisms
T	218	3.08	Signal transduction mechanisms
M	270	3.81	Cell wall/membrane biogenesis
N	64	0.90	Cell motility
U	79	1.12	Intracellular trafficking and secretion
O	121	1.71	Posttranslational modification, protein turnover, chaperones
C	214	3.02	Energy production and conversion
G	263	3.71	Carbohydrate transport and metabolism
E	420	5.93	Amino acid transport and metabolism
F	128	1.81	Nucleotide transport and metabolism
H	177	2.50	Coenzyme transport and metabolism
I	129	1.82	Lipid transport and metabolism
P	243	3.43	Inorganic ion transport and metabolism
Q	83	1.17	Secondary metabolites biosynthesis, transport and catabolism
R	653	9.22	General function prediction only
S	505	7.13	Function unknown
-	1978	27.9	Not in COGs

^a^The total number is based on the total number of protein coding genes in the genome

## Insights from the genome sequence

To investigate the phylogeny of 10.1601/nm.5000 MYBT18246 two approaches were used. First, nineteen 10.1601/nm.4857 strains were chosen for 16S rRNA analysis within the 10.1601/nm.4857 clade (Fig. 2a). The 16S rRNA phylogeny shows that 10.1601/nm.5000 MYBT18246 clusters with other Bcsl group members within the 10.1601/nm.4857 clade. However, the low bootstrap values confirm the limitations of 16S rRNA as a discriminatory marker within the Bcsl species group. Second, we applied an MLST approach based on the scheme by Priest et al. [36]. This revealed that MYBT18246 clusters with the toxin cured 10.1601/nm.5000 Bt407, insecticidal 10.1601/nm.5000 serovar *chinensis* CT-43, and with the nematicidal 10.1601/nm.5000 YBT-1518 within the Bcsl phylogeny (Fig. 2b). Based on this phylogeny and the phenotypic defining feature of the 10.1601/nm.5000 species group (the ability to produce crystal toxins against invertebrates and nematodes), the strain 10.1601/nm.5000 MYBT18246 can be safely classified as nematicidal 10.1601/nm.5000.

For a detailed analysis of encoded toxins in 10.1601/nm.5000 MYBT18246, we generated a local database consisting of all available Cry, Cyt, Vip and Sip protein sequences from UniProtKB [71] and GenBank [72]. The database was curated to generate a set of non-redundant reference toxins. In total, we identified three different *cry* toxin genes in the 10.1601/nm.5000 MYBT18246 genome and classified them as *cry*13Aa2 (>95%), *cry*13Ba1 (<78%) and *cry*13Ab1 (<95%), based on the similarity scheme from the Cry-toxin naming committee by Crickmore [13, 70]. Notably, these *cry* toxin genes are encoded on the chromosome and not on extra-chromosomal elements as has been previously reported for the vast majority of *cry* toxin genes [7, 73, 74]. The toxin gene analysis revealed four additional putative toxin-like genes on plasmids with sequence similarity to *cry* genes and *vip* genes. A Pfam domain analysis using InterPro [75] revealed a p120510 encoded putative Vip-like toxin, a p120416 encoded putative Cry-like toxin and two p109822 encoded putative Bin-like toxins with potential for future studies.

**Fig. 3:**
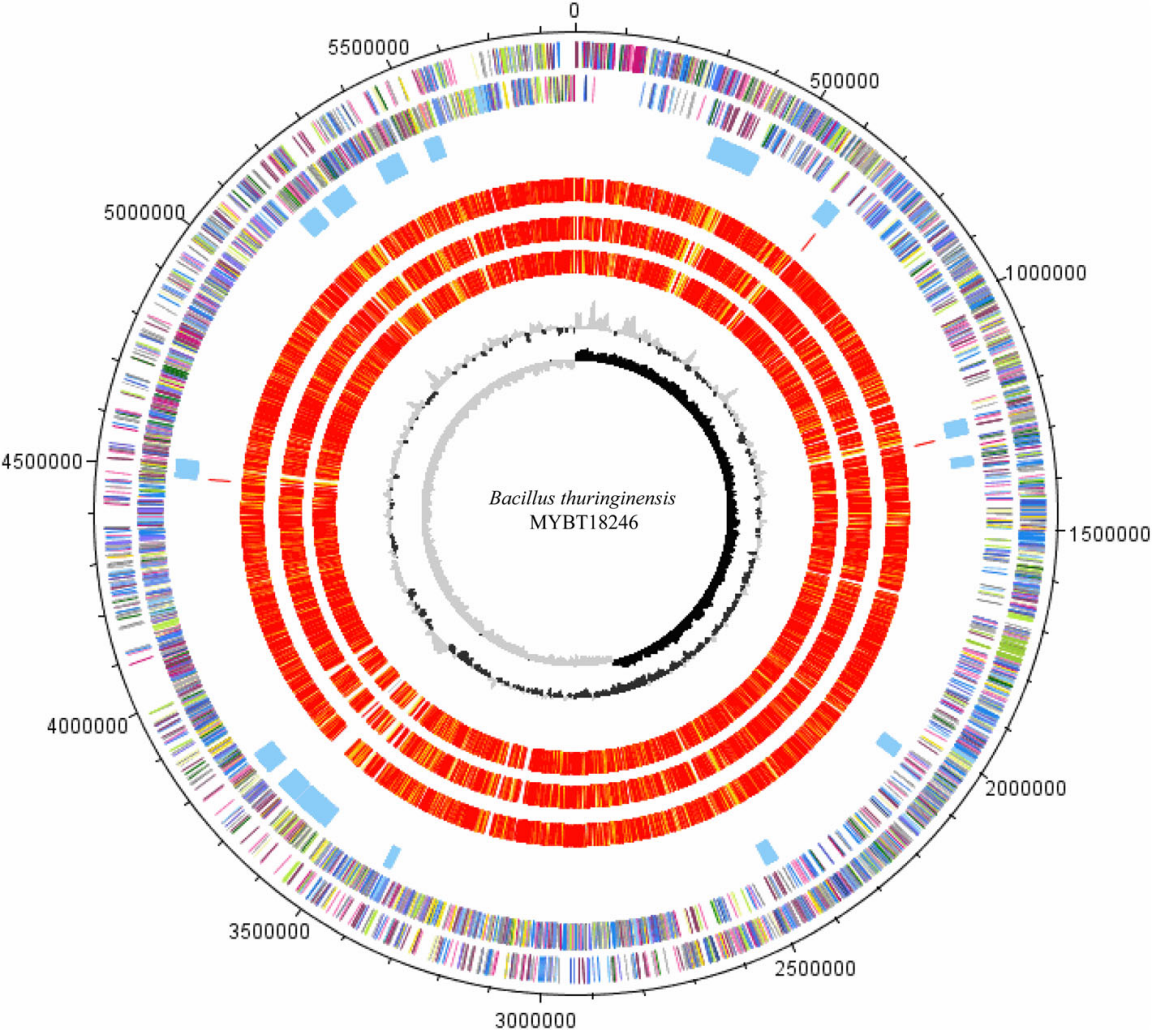
Circular visualization of the genome comparison of 10.1601/nm.5000 MYBT18246 with 3 other sequenced 10.1601/nm.5000 strains. The tracks from the outside represent: (track 1–2) Genes encoded by the leading and lagging strand of 10.1601/nm.5000 MYBT18246 marked in COG colors [93]; (track 3) putative prophage regions, identified with PHAST in blue [76], (track 4) identified *cry* toxin genes in red; (track 5–7) orthologs for the genomes of 10.1601/nm.5000 YBT-1518 (CP005935.1), 10.1601/nm.5000 CT-43 (CP001907.1), 10.1601/nm.5000 Bt407 (CP003889.1) illustrated in red to light yellow, singletons in grey (grey: <1e^−20^; light yellow: 1e^−21^–1e^−50^; gold: 1e^−51^–1e^−90^; light orange: 1e^−91^–1e^−100^; orange: 1e^−101^–1e^−120^; red: >1e^−121^ (track 7) % GC plot (track 8), GC skew [(GC)/(G + C)]. Visualization was done with DNAPlotter [94]

Additionally, the 10.1601/nm.5000 MYBT18246 chromosome was screened for prophage regions by using the Phage Search Tool with default parameters. PHAST identifies prophage regions based on key genes from a reference database and defines the boundaries using a genomic composition-based algorithm. For a more detailed description see [76]. A total of 16 putative prophage loci were identified in the chromosome, including three that were associated with the previously identified chromosomally encoded *cry* toxin genes. As shown in Fig. 3, the *cry* toxins (displayed in red, track 4) are located in close proximity to identified prophage regions (displayed in blue, track 3). Furthermore, all 10.1601/nm.5000 MYBT18246 extra-chromosomal elements were also screened for prophages to check whether we could identify phages that reside in a linear or circular state in the host, as has been reported in 2013 by Fortier et al. [77]. Apparently, intact phage regions were identified according to the PHAST score system on p150790, p120416, p109822, p101287 and p46701.

The finding of prophage associated *cry* genes in strain MYBT18246 indicates that phages may serve as vectors for the transmission of virulence factors within the species 10.1601/nm.5000 *.* This resembles the previously described lysogenic conversion of pathogens by phages [78], supporting the idea that phages may represent a driving force for the distribution of fitness factors as well as virulence factors [78–80]. The finding that toxins, which are generally specific for a certain type of host organism, are located within a mobile genomic element in the chromosome of this bacterium, suggests that phages of strain MYBT18246 may contribute to adaptation to different hosts [81–83].

## Extended insights

**Fig. 4:**
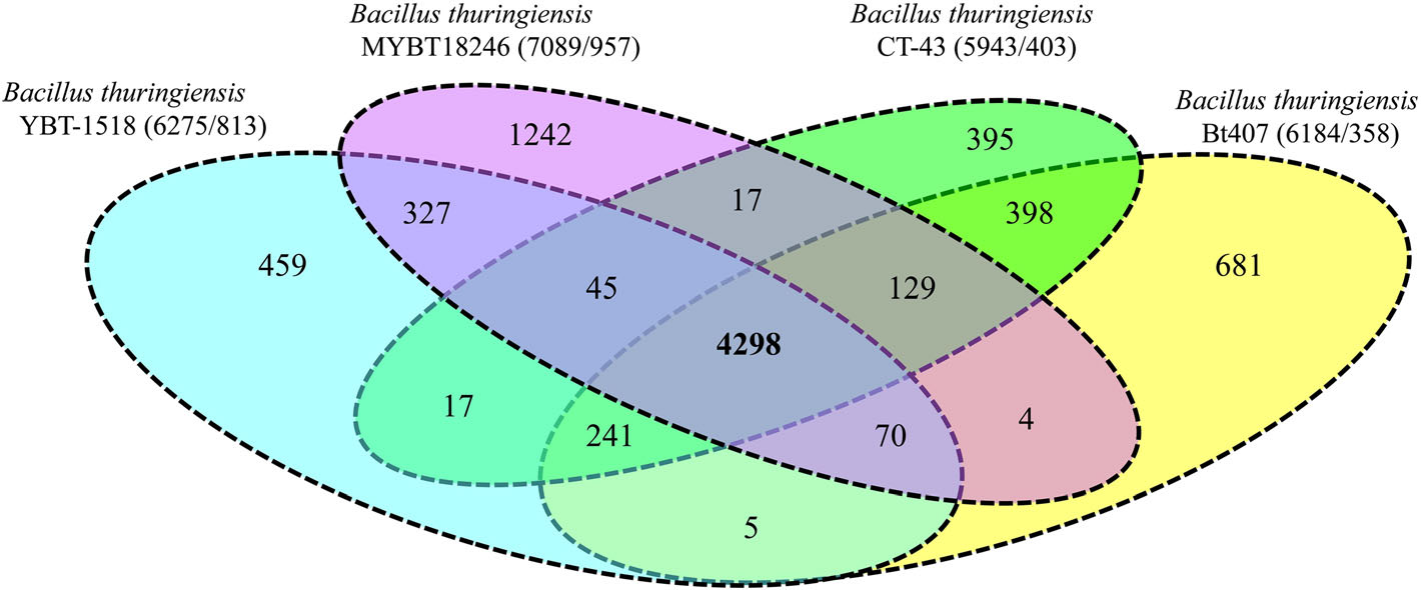
Venn diagram of the genome comparison of 10.1601/nm.5000 MYBT18246 with other 10.1601/nm.5000 strains. Venn diagram displays the orthologous genes between 10.1601/nm.5000 MYBT18246 (CP015350-CP015361), 10.1601/nm.5000 YBT-1518 (CP005935-CP002486), 10.1601/nm.5000 serovar *chinensis* CT-43 (CP001907-CP001917) and 10.1601/nm.5000 Bt407 (CP003889-CP003898). Ortholog detection was performed with Proteinortho [84] including protein blast with a similarity cut-off of (50%) and an E-value of 1e^−10^. The total number of genes and paralogs are depicted under the corresponding species name. Open reading frames that were classified as pseudogenes were not included in this analysis

Based on the proximity within the tree (Fig. 2b), the genomes of 10.1601/nm.5000 Bt407, 10.1601/nm.5000 serovar *chinensis* CT-43 and 10.1601/nm.5000 YBT-1518 were identified as closest relatives and selected for an in depth comparative analysis. Shared gene contents were determined, visualized and compared, with a focus on known virulence factors such as *cry* toxins and pathogenic driving forces such as phages. The analysis revealed unique as well as shared gene contents for each strain (Fig. 3). In Fig. 3 the outer rings represent the genes on the leading and lagging strand with COG classification. The inner rings (track 5–7) illustrate the orthologous genes of 10.1601/nm.5000 YBT-1518, 10.1601/nm.5000 CT-43, 10.1601/nm.5000 Bt407 in red (high similarity) to light yellow (low similarity), and white (no similarity). The circular representation of the chromosome comparison revealed that prophages are a major source of regional differences between the strains (Fig. 3). Additionally, the pan-genome of 10.1601/nm.5000 MYBT18246 compared to the three closest relatives was determined (Fig. 4). Orthologous genes between all four organisms were identified by comparing the whole genomes using Proteinortho [84] with a similarity cutoff of 50% and an E-value of 1e^−10^. Gbk-files were downloaded from NCBI and the protein sequences were extracted using cds_extractor v0.7.1 [85]. Detected paralogous genes are displayed in the Venn diagram in Fig. 4. All four strains share a core genome of 4298 genes. This is equivalent to 67% of each genome. 10.1601/nm.5000 MYBT18246 shares 4 additional genes exclusively with 10.1601/nm.5000 Bt407, 17 genes with 10.1601/nm.5000 serovar *chinensis* CT-43 and 327 genes with 10.1601/nm.5000 YBT-1518. 10.1601/nm.5000 serovar *chinensis* CT-43 and 10.1601/nm.5000 Bt407 share 398 orthologous genes. Notably, the genome of 10.1601/nm.5000 MYBT18246 contains 1242 orphan genes and thus two to threefold more singletons than the compared genomes. This result confirms the high degree of conservation of the four 10.1601/nm.5000 strains (Fig. 2a and b) and it also refines the phylogenetic relationship of the strains to each other based on non-orthologous regions. Singletons are located on the chromosome as well as on extra-chromosomal elements. The density of singletons is higher (2.5 fold) on the plasmids. Notably, all major chromosomal differences can be attributed to prophage regions. All gene products were assigned to COG categories and investigated for PFAM domains and Signal peptides (Table 6). In detail, those genes code for: (i) phage proteins, (ii) morons (virulence factors), (iii) a vast majority of proteins with cryptic function. This is supported by Fig. 3 which clearly shows that the regions of differences (track 5–7) directly correspond to the regions of identified phages (track 3). Moreover, the identified *cry* toxins (track 4) are adjacent to identified prophage regions and could be suggested as morons. Additionally, the singletons were screened for further virulence factors and genes encoding type-IV secretion system, C5-methyltransferase, type-restriction enzymes, sporulation, resistance and genes involved in genetic competence were identified. In particular, the finding of restriction-modification systems indicates a protection mechanism against other phages and plasmids and thus forms a putative barrier against further genomic modification.

**Table 6:** General genome features of 10.1601/nm.5000 MYBT18246 and close relatives

Genome features	Genome name			
	10.1601/nm.5000 MYBT18246^a^	10.1601/nm.5000 407^b^	10.1601/nm.5000 YBT-1518^c^	10.1601/nm.5000 CT-43^d^
Sequencing status	Finished	Finished	Finished	Finished
Genome size (Mbp)	6.75	6.13	6.67	6.15
DNA coding (bp)	5,623,665	5,133,026	5,421,574	5,079,667
GC (%)	35.4	35.02	35.29	35.12
DNA scaffolds	12	10	7	11
Total gene count	7239	6442	6738	6252
Protein coding genes (%)	97.9	95.9	98.0	95.1
RNA genes	151	180	139	124
Genes in internal clusters	2694	489	370	334
Genes with function prediction	5274	4615	5193	4211
Genes assigned to COGs	4662	3634	3746	3505
Genes with Pfam domains	5503	4991	5333	4809
Genes with signal peptides	500	447	471	418
Genes with transmembrane helices	1863	1750	1854	1698
CRISPR repeats	0	2	0	2

Accession numbers: ^a^CP015350, ^b^CP003889, ^c^CP005935, ^d^CP001907

## Conclusion

In this work we present the whole-genome sequence of 10.1601/nm.5000 MYBT18246 and its specific genome features. The genome includes three nematicidal *cry*13 gene variants located on the chromosome, which were named according to sequence similarity as stated by the Cry Toxin Nomenclature Committee, as *cry*13Aa2, *cry*13Ba1, and *cry*13Ab1. Four additional putative toxin genes were identified with low sequence similarity to other known toxins on plasmids: p120510 (Vip-like toxin), p120416 (Cry-like toxin) and p109822 (two Bin-like toxins). These toxins contained complete toxin domains, yet the activity against potential hosts should be elucidated in future studies. The genome comprises a large number of mobile elements involved in genome plasticity including eleven plasmids and sixteen chromosomal prophages. Both plasmids and prophages are important HGT elements indicating that they are an important driving force for the evolution of pathogens. The most striking finding is the close proximity of the chromosomal nematicidal *cry* toxin genes to three distinct prophages indicating a contribution of phages in defining the host range of this strain. 10.1601/nm.5000 MYBT18246 may show potential as a biocontrol agent against nematodes which should be addressed in future experiments.

## Abbreviation

*B*: 10.1601/nm.4857
*B. thuringiensis*: 10.1601/nm.5000
Bcsl: 10.1601/nm.4885 sensu *lato*
Cry: Crystal
Cyt: Cytolytic
IMG: Integrated Microbial Genomes
MLST: Multi-locus sequence typing
PHAST: Phage Search Tool
Sip: Secreted insecticidal protein
SMRT: Single molecule real-time
Vip: Vegetative insectididal protein
